# Fracture resistance to treated teeth using known endodontics techniques in Indian patients

**DOI:** 10.6026/97320630018184

**Published:** 2022-03-31

**Authors:** Renu Batra, Mallwika Sisodiya, Puja Kumari, Sunpreet Kaur, Prajakta B Patil, Shresth Kumar Bhagat

**Affiliations:** 1 Department of Conservative Dentistry and Endodontics, NIMS Dental College, Jaipur-302002, Rajasthan, India; 2 Department of Conservative Dentistry and Endodontics, Mithila Minority Dental College & Hospital, Darbanga-846003, Bihar, India; 3 PG Student, Mithila Minority Dental College, Darbanga & Hospital (MMDCH), Darbanga-846003,, Bihar, India; 4 MM College of Dental Sciences and Research, Mullana-133203, Haryana, India; 5 Department of Conservative Dentistry and Endodontics, Bharati Vidyapeeth Dental College & Hospital, Sangli-416406, Maharastra, India; 6 Department of Conservative Dentistry, Endodontics and Aesthetic Dentistry, Sarjug Dental College, Darbhanga-846001, India

**Keywords:** Fibre reinforced posts, Fracture, Quartz post

## Abstract

Teeth with crown structure less than 50% can be restored. Therefore, it is of interest to evaluate an in vitro efficacy of Zirconia post, Glass fiber post, polyethylene-woven fiber posts, and Quartz posts. Forty eight recently extracted mandibular first premolar teeth were randomly grouped in to 4 different groups with 12 samples in each group. After endodontic treatment samples in all groups underwent post preparation followed by restoration with respective posts. The mean fracture resistance (Newton) were 463.5 ± 14.3 (Group I) 425.2± 23.5 (group II), 410.4± 18.6 (Group 3) and 385.2 ± 14.2 (group 4). Data shows that Zirconia post had highest fracture resistance compared to other tested groups.

## Background:

Esthetics and structural durability plays a major role in treatment outcome of endodontically treated teeth. There has been an increased demand for endodontic post and core materials for destructed endodotically treated teeth [1]. Teeth with crown structure less than 50% can be restored with post and core. It is widely used to sustain the prosthetic crown and main aim is to maintain retentive and resistant form [2]. There are numerous posts available in the market. Metal posts possess higher hardness compared to fiber posts. Therefore, fiber posts facilitate more suitable stress distribution in the root. Metal posts are more vulnerable to fracture than fiber posts. Fiber-reinforced composite (FRC) posts being identical hardness number to that of dentin and thus have optimal toughness [3]. Various types of esthetic posts are available in the market such as carbon, ever Stick (E-glass), glass fiber and fiber reinforced and quartz posts [4-6]. Therefore, it is of interest to evaluate an in vitro efficacy of Zirconia post, Glass fiber post, polyethylene-woven fiber posts, and Quartz posts.

## Materials & Methods:

This study was conducted in the department of Cons Dent & Endodontics. This study was commenced after obtaining approval from ethical committee of institute in which 48 recently extracted mandibular first premolar due to orthodontic purpose was taken. We randomly grouped 48 teeth into 4 different groups with 12 samples in each groups; Group I: Zirconia post, Group II: Glass fiber post, Group III: polyethylene-woven fiber posts, Group IV: Quartz post. Samples in all groups underwent endodontic treatment following all standardized parameters with the step-back technique. Following root canal treatment, silicone impression material was applied on all roots to simulate the periodontal ligament and was mounted in cubic acrylic molds. Post space preparation was done using peeso reamers (Mani, Tochigi-ken, Japan).In all cases three mm of the post was extended above the cementoenamel junction and post were cemented into the prepared post space with cements. Based on manufacturer's instructions, intra canal posts were cemented into the canal using Panavia F2 resin cement. Over coronal extension of post, core built up was done to receive crown. The crowns were seated on the teeth in each group. Using universal testing machine, fracture resistance was calculated by applying load at 45° angle to long axis of the tooth at a crosshead speed of 1 mm/min. The point where fracture occurred was recorded with the formula, shear bond strength (MPa) = Load (N)/Surface area (mm2). A descriptive statistic was applied based on SPSS version 21.0 (USA). One way analysis of variance and pos hoc Bonfeeri test was used for comparison between groups and the level of significance was set below 0.05.

## Results:

Table 1(see PDF) indicates distribution of teeth based on type of core material and post used. Each group comprised of 10 teeth. Table 2(see PDF), indicate mean fracture resistance (Newton) in group I was 463.5 ± 14.3, in group II was 425.2 ± 23.5, in group III was 410.4 ± 18.6, in group IV was 385.2 ± 14.2. Table 3(see PDF) indicates intra group comparison of mean fracture resistance of different core materials. The mean variation with group I over group II, Group III Group IV was 38.3, 53.1 and 78.3 respectively. Group vs group III and Group IV was 14.8 and 40 respectively. Group III vs Group IV was 25.2. Group I zirconia had highest fracture resitsance compared to other tested groups. The difference was significant (P<0.05).

## Discussion:

Endodontically treated teeth are usually restored with posts when the remaining tooth structure cannot provide sufficient support and retention for restoration. Restoring these teeth using materials with a comparable elastic modulus to dentine are advantageous due to the decreased risk of root fracture [6]. In this study we compared efficacy of Zirconia post, Glass fiber post, polyethylene-woven fiber posts, and Quartz post. From this study we found that maximum fracture resistance was observed with zirconia posts. Pruthi et al. (2018) assessed the fracture resistance of different fiber reinforced posts and concluded that, parallel posts to have better retention than tapered and double tapered posts [1]. Saritha et al. (2017) evaluated the fracture resistance of carbon, zirconia and glass fiber post and found that zirconia had good fracture resitance similar to our results [5]. Izadi et al. (2020) took 108 teeth and evaluated fracture resistance of endodontically treated teeth based on of three core building materials with fiber reinforced composite (FRC) posts and ParaPosts. It was found that maximum fracture resistance (423.7 ± 111.7) was seen with FRC posts + Core Max II with bonding agent while minimum (242.3±73.4) was seen with ParaPosts + LuxaCore. There was no significant difference with the fracture resistance of other groups (P > 0.05) [7]. Torabi K & Fattahi F (2009) compared the fracture resistance of different fibers reinforced with composite posts and concluded that variation in FRC posts did not offer any significant variation in the load failure and the mode of fracture [8]. FRC posts such as prefabricated glass, carbon fiber posts, quarz-fiber posts; Individual glass fiber posts, polyethylene fiber posts and hollow fiber posts are usually indicated in caries, fractured or traumatized teeth [9]. These have low elastic modulus (18-42 GPa), which is similar to that of dentine, nontoxic, chemically inert and excellent light conductivity (11mm). Apart from their advantages, there are few disadvantages of these posts such as these cannot be used in teeth with failed root canal treatment, teeth with poor prognosis, teeth having fragile roots and increased mobility [10]. In contrast to our finding Sharma et al (2016), evaluated the fracture resistance of glass fiber post with carbon and quartz post and concluded that quartz post had higher fracture resistance compared to other groups [11]. Some critical factor that can impact the fracture resistance and prognosis of an endodontically treated tooth such as; ferrule effect, the type of core material used and design of the post. LuxaCore, a dual-cured core buildup material was used in our study since it has ability to bond to both the glass fiber post and tooth structure. Bonding between the fiber post and the root dentin improves stress distribution. The choice of the posts is predetermined to the dimension of the root canal and restricted by the root length [12]. Balkaya et al (2021) assessed the effectiveness of various coronal restorations on the fracture resistance of immature teeth and concluded that Ribbond in combination with composite resin enhances the fracture resistance of teeth [13]. Mello et al (2020) evaluated the fracture resistance of endodontically treated teeth with either composite restoration in the crown area, composite restoration in the crown and 3 mm into the root, composite restoration in the crown area, revascularization with a composite in the crown area and control group. They found that there were no statistically significant differences in the fracture resistance among the 5 groups [14]. Sogukpinar A, Arikan (2020) concluded from their study that, fracture resistance was significantly higher at the two week and the two-month mark compared to the one-year mark for each biomaterials [15]. Ali et al (2019) assessed the fracture resistance of immature teeth after regenerative endodontic procedure. They stated that all treated teeth showed considerably lesser resistance to fracture in comparison to the intact teeth [16]. Silva et al (2018) from a systematic review on fracture resistance of endodontically treated teeth found that, there is no evidence that supports the use of contracted endodontic cavities over traditional endodontic cavities for the increase of fracture resistance in human teeth [17]. Li et al (2011) evaluated the effect of various posts on the fracture resistance of endodontically-treated immature teeth. They concluded that teeth restored with fiber posts are more resistant to fracture than those restored with either metal posts or non-post [18]. The limitation of present study is lesser sample size. The variation if results may be due to type of tooth selected, amount and direction of force applied and root morphology, this study could not reproduce the oral conditions since it was in vitro study, in contrast to masticatory forces in the mouth, samples were tested under static load. The samples were not exposed to ageing, fatigue loading and thermal cycling, further in vitro studies required with larger samples size and with thermal cycling and fatigue loading for evaluation.

## Conclusion:

We show that FRC posts showed higher fracture resistance as compared to FRC para posts. Moreover, fracture resistance was not dependent on type of material used.
